# Advances of Antimicrobial Peptide‐Based Biomaterials for the Treatment of Bacterial Infections

**DOI:** 10.1002/advs.202206602

**Published:** 2023-02-01

**Authors:** Guoyu Li, Zhenheng Lai, Anshan Shan

**Affiliations:** ^1^ The Institute of Animal Nutrition Northeast Agricultural University Harbin 150030 P. R. China

**Keywords:** antimicrobial peptides, bacterial infection, biomaterial delivery, design strategy, mouse models, treatment potential

## Abstract

Owing to the increase in multidrug‐resistant bacterial isolates in hospitals globally and the lack of truly effective antimicrobial agents, antibiotic resistant bacterial infections have increased substantially. There is thus an urgent need to develop new antimicrobial drugs and their related formulations. In recent years, natural antimicrobial peptides (AMPs), AMP optimization, self‐assembled AMPs, AMP hydrogels, and biomaterial‐assisted delivery of AMPs have shown great potential in the treatment of bacterial infections. In this review, it is focused on the development prospects and shortcomings of various AMP‐based biomaterials for treating animal model infections, such as abdominal, skin, and eye infections. It is hoped that this review will inspire further innovations in the design of AMP‐based biomaterials for the treatment of bacterial infections and accelerate their commercialization.

## Graphical abstract

Owing to the increase in drug‐resistant bacteria and the lack of truly effective bacteriostatic agents, it is urgent to explore and design new nonantibiotic bacteriostatic agents with potential. Antimicrobial peptide (AMP)‐based biomaterials are explored as antibacterial agents for treating infections. This article focuses on the therapeutic efficacy and potential of various AMP‐based biomaterials in animal models of bacterial infections.

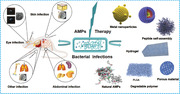

## 1 Introduction

The discovery of penicillin in the 20th century provided a transformative advantage in the fight against bacterial infections. However, bacterial evolutions resulting from the ill‐advised high‐dose use of antibiotics has led to antibiotic resistance. In 2019, three bacterial infection syndromes (respiratory and chest, bloodstream, and intra‐abdominal infections) attributable to antimicrobial resistance (AMR) contributed the most to the global burden. Six pathogens were responsible for more than 250 000 deaths associated with AMR: *Escherichia coli*, *Staphylococcus aureus*, *Klebsiella pneumoniae*, *Streptococcus* *pneumoniae*, *Acinetobacter baumannii*, *and Pseudomonas aeruginosa*.^[^ ^1^ ^]^ Recent estimates suggest that by 2050, 10 million people will die annually from drug‐resistant bacterial infections.^[^ ^2^ ^]^ Antimicrobial treatments currently being developed are inadequate to address the growing threat of antibiotic resistance, according to the annual pipeline report by the World Health Organization. Therefore, the research and development of new antibacterial agents have attracted increasing attention.^[^ ^3^ ^]^

Antimicrobial peptides (AMPs) have gained attention as potential alternatives to traditional antibiotics owing to their broad antimicrobial activity, high specificity, and ability to modulate host immunity.^[^ ^4^ ^]^ Currently, AMPs have been explored as an alternative to antibiotic therapy for treating bacterial infections. This alternative therapy is superior in the treatment of bacterial infections (**Table** **1**). For example, AMP PL‐5 is indicated for the treatment of bacterial infections of the skin and wounds, particularly those caused by stubborn, drug‐resistant bacteria. The AMP PL‐5 spray was clinically determined to be a safe and effective treatment for skin wound infections.^[^ ^5^ ^]^ Similarly, human lactoferrin N‐terminal 11‐peptide (hLF1‐11) has been shown to be effective in animal models of osteomyelitis and other bacterial infections, with significant efficacy observed in phase I trials.^[^ ^6^ ^]^ AMPs have been shown to have good antibacterial properties. However, some AMPs have limited stability under physiological conditions, challenges in delivery to the site of infection, and it is difficult to design formulations while maintaining the activity of these drugs. Therefore, the clinical application of AMPs to treat bacterial infections remains limited.^[^ ^7^ ^]^

**Table 1:** AMPs in clinical trials to treat bacterial infections

Infection type	DRAMP ID^a)^	Name	Bacteria	Medical use	Stage of development
Abdominal infection	DRAMP18080	Plectasin	*Pneumococcal* and *Streptococcal infections*	Systemic treat Gram positive, especially *pneumococcal* and *streptococcal* infections	Phase I
	DRAMP18068	hLF1‐11	*Methicillin‐resistant S*. *aureus* (MRSA)	LPS‐mediated diseases and fungal infections	Phase I(Completed)
Skin, wound infection	DRAMP28983	PL‐5	*Pseudomonas aeruginosa*, *and* MRSA and *Multidrug resistant A*. *baumannii* containing NDM‐1 gene	Skin wound infection	Phase II
	DRAMP18160	Omiganan (MBI 226/MX‐226/CLS001)	*Staphylococci*: MSSA, MRSA, MSSE, and MRSE	Treatment of atopic dermatitis (AD), usual type vulval intraepithelial neoplasia (uVIN), external genital warts, acne vulgaris, rosacea, and facial seborrheic dermatitis	Phase III (Failure), Phase III (rosacea, completed), Phase II (AD), uVIN external genital warts, and acne vulgaris, completed), Phase III (facial seborrheic dermatitis, recruiting)
	DRAMP18158	PMX 30 063 (brilacidin)	*Staphylococcusspp*	Acute bacterial skin infections caused by *Staphylococcusspp*	Phase II
	DRAMP18154	XOMA‐629	*S. aureus*	Impetigo	Phase IIA
	DRAMP18157	Novexatin (NP213)	—	Treatment of dermatophyte fungal infections	Phase IIb
	DRAMP18057	MSI‐78(Pexiganan)	*Broad‐spectrum* antibacterial activity	Impetigo	Phase III (Failure)
Lung infection	DRAMP18060	Iseganan(IB‐367)	*Broad‐spectrum* antibacterial activity	An aerosolized treatment for ventilator‐associated pneumonia	Phase III (Failure)
	DRAMP20774	Murepavadin (POL7080)	*K. pneumoniae*, *E. coli*, *A. baumannii*	Treatment of nosocomial pneumonia and ventilator‐associated bacterial pneumonia.	Phase III
	DRAMP18163	Ghrelin	—	Airway inflammation, chronic respiratory infection and cystic fibrosis	Phase II (Completed)
Oral infection	DRAMP20760	C16G2	Gram‐positive bacteria	Treatment of adult and adolescent dental subjects	Phase II
	DRAMP18081	PAC113	Fungus	Oral candidiasis	Phase IIb
	DRAMP18059	Iseganan (IB‐367)	Broad‐spectrum antibacterial activity	A mouth rinse to prevent polymicrobial infection associated with oral mucositis in patients receiving chemotherapy	Phase III (Failure)
	DRAMP18062	Histatin	Fungus	Chronic Pseudomonas aeruginosa infections	Phase I
	DRAMP18061	Histatin	Fungus	AMPs‐containing mouth wash for the treatment of oral candidiasis (gingivitis and periodontal diseases)	Phase II‐III
Other bacterial infections	DRAMP20761	LTX‐10 9	MRSA	Treatment of Nasal Carriers MRSA	Phase I/IIa
	DRAMP18152	IMX942	—	Nosocomial infections, Febrile, Neutropenia	Phase II
	DRAMP18069	rBPI21(Neuprex)	—	Meningococcaemia; prophylactic treatment of infectious complications from post‐traumatic bleeding	Phase III (Failure)
	DRAMP18083	CZEN‐002	Candida	Vulvovaginal candidiasis	Phase IIb
	DRAMP18161	OP‐145	—	Chronic bacterial middle ear infection	Phase II (Completed)

^a)^ Database: DRAMP. http://dramp.cpu‐bioinfor.org/.AMPs for treating different bacterial infections were screened and classified based on the antimicrobial peptides used in clinical trials from the database.

**Figure 1:**
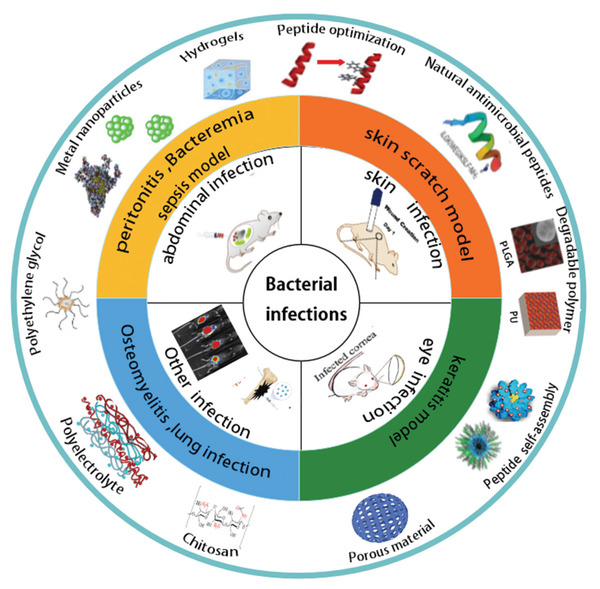
In the AMP studies, four types of mouse models were established according to the different sites of bacteria infection, and various types of AMP‐based biomaterial therapies.

In vitro studies of mature AMPs must be conducted in vivo to enable the clinical advancement of peptides.^[^ ^8^ ^]^ The in vivo experimental models of AMPs can be divided into four main categories: abdominal infection model represented by peritonitis, skin infection model represented by skin scratches, eye infection model represented by keratitis, and other infection models represented by osteomyelitis and lung infection (**Figure** **1**). This review builds on these studies for the treatment of bacterial infections and describes the therapeutic potential of AMP‐based therapies, which include natural AMPs, AMP optimization strategies, AMP and biomaterial delivery systems, and AMP and nanoparticle noncovalent conjugation approaches. The focus was on the therapeutic efficacy and potential.

Previous reviews have focused on the optimal design of AMPs in terms of activity or stability, while there has been a lack of evaluation of their therapeutic effects in animal models. Therefore, in this review, we comprehensively summarize the design and optimization strategies of AMP‐based biomaterials for therapeutic efficacy in vivo in four major infection models and discuss their development and biological advantages. Finally, we discuss the current barriers and future challenges for AMP‐based biomaterials.

## 2 Abdominal Infection

Bacterial peritonitis is a common clinical complication of abdominal surgery, trauma, or intra‐abdominal infection, which can cause multisystem failure and eventually death. The efficacy of this model can provide theoretical and experimental basis for clinical treatment.^[^ ^9^ ^]^ A mouse model of peritonitis is often used to assess the potential of AMPs to treat bacterial infections in vivo.^[^ ^10^ ^]^ Commonly used bacteria include *S. aureus*, *E. coli*, *K. pneumoniae*, and MRSA. These models are broadly classified into two types depending on the injection site. 1) Intraperitoneal inoculation and tail vein injection: the minimum lethal dose of bacteria was first determined by the survival rate of mice 48 h after attack with different doses of bacteria. At this lethal dose, 100% of mice died within 48 h of infection. Treatment was administered by intraperitoneal injection of AMPs into the tail vein 1 and 5 h after infection with this dose of bacteria. 2) Inoculation and administration by intraperitoneal injection: this included intraperitoneal injection of bacteria for infection, followed by intraperitoneal injection of AMPs 1 h later. The different articles had slight differences in model building, mainly in the time of infection and the duration and number of peptide treatments. However, the indicators used to evaluate the effects of the peptide treatment were generally the same. The main indicators were the bacterial load in the liver, spleen, lungs, and kidneys, the level of inflammatory factors in the blood of the mice, organ tissue section observation, and the survival rate of the mice.

### 2.1 Natural AMP Therapy

**Figure 2:**
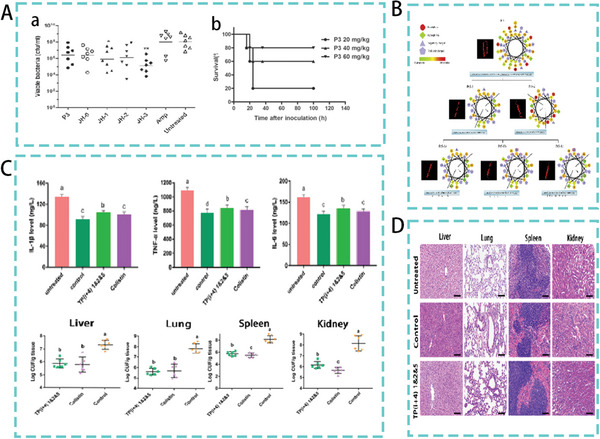
A) (a) Bacterial load in the blood of mice. (b) Survival of mice after treatment with different concentrations of P3. Reproduced with permission.^[^ ^13^ ^]^ Copyright 2015, American Society for Microbiology. B) Schematic diagram of Pt‐derived peptide substitution. Reproduced with permission.^[^ ^19^ ^]^ Copyright 2018, Academic Press. C,D) Therapeutic effect of TP(i+4)1&2&5 in a mouse model of peritonitis. Reproduced under the terms of the CC BY 4.0 license.^[^ ^20^ ^]^ Copyright 2022, Frontiers Media S.A.

The discovery of natural AMPs has substantially enriched the field of peptide antibacterial drugs.^[^ ^11^ ^]^ Hu et al. identified a novel 18‐residue linear AMP, P3, derived from the central portion of the alpha subunit of bovine hemoglobin.^[^ ^12^ ^]^ In a subsequent study, the therapeutic effect of P3 was measured in a mouse model of bacteremia. The results showed that although the P3 treatment group did not significantly reduce the number of bacteria in the blood of the mice, a survival rate of 80% was achieved 100 h after treatment with a high dose of P3 (60 mg kg^−1^) (**Figure** **2**A).^[^ ^13^ ^]^ Although P3 showed efficient bactericidal activity in vitro, it was not significantly effective in vivo. This may be related to the poor stability of the natural peptide in vivo, where the presence of proteases predisposes the peptide to inactivity owing to the presence of enzyme sites. However, natural peptides are also potential human therapeutic agents. For example, plectasin, the first defensin isolated from a fungus, caused a 10‐ and 1000‐fold decrease in the concentration of viable *Pneumococcal* in the peritoneum at 2 and 5 h, respectively, in a model of peritonitis induced by three *Pneumococcal* strains. Survival data showed that the treatment rate was as high as 100% for total cure, and the cure was as effective as vancomycin and penicillin.^[^ ^14^ ^]^ However, compared to AMPs that bind to cell membranes and directly disrupt membrane function, plectasin works by directly binding to bacterial cell wall precursor lipid II, which has the potential to induce bacterial resistance.^[^ ^15^ ^]^ Therefore, the mechanism of action and application of plectasin needs to be further investigated and evaluated. Overall, although AMPs have some potential for treating abdominal infections, they also face many challenges, such as poor protease stability,^[^ ^16^ ^]^ high toxicity, and side effects. In this context, research on the optimization of natural AMPs continues to expand.

### 2.2 Optimized Design Based on Natural AMP Sequences

In recent years, optimization and modification of natural AMPs have improved their clinical applications. The modifications mainly focused on improving the activity and stability of AMPs.^[^ ^17^ ^]^ We roughly divided common modifications into two categories. The first is the modification strategy based on natural amino acids,^[^ ^18^ ^]^ such as amino acid‐directed mutagenesis, database‐based screening and design, targeted antimicrobial peptide design, novel amphiphilic peptide templates, and enzyme‐resistant peptide design. The other category is modification strategies using unnatural amino acids, such as fatty acid modification and D‐amino acid modification.

#### 2.2.1 Amino Acid Substitution

Site‐directed optimization design involves reoptimization of native peptides by the addition, deletion, or strategic substitution of one or a few amino acid residues. This method effectively improved the activity of the original peptide. For example, Wang et al. designed a new AMP by shortening the sequence and replacing the original peptide Pt5 with Trp (W) and Lys (K) at selected positions (Figure 2B). The optimized derivative peptide, Pt5‐1c, showed the most potent antibacterial activity with a minimum inhibitory concentration (MIC) of 1.2–4.8 µm.^[^ ^19^ ^]^ Similarly, our previous study, based on the natural AMP TP, used Lys and Trp systematically to replace paired common amino acids in hydrogen bond forming positions. Engineered synthetic TP(i+4)1&2&5 exhibited the highest activity in vitro (MIC = 2 µm), and in vivo therapeutic potential of the optimized peptide was subsequently assessed in an *E. coli*‐mediated peritonitis test in mice. As shown in Figure 2C,D, TP(i+4)1&2&5 treatment showed groups significantly reduced bacterial load in mouse organs compared to the infected group. Analysis of the inflammatory factor levels in the blood showed that TP(i+4)1&2&5 significantly reduced inflammatory factor levels and had a potential anti‐inflammatory effect. Histological sections further confirmed the effect of the optimized peptide in alleviating tissue damage.^[^ ^20^ ^]^ The in vitro antibacterial activity of the peptide can be significantly improved by site‐directed replacement of amino acids, but the therapeutic effect in vivo was found to be unstable. In terms of mouse survival data, optimized AMP was less effective than peptides with high protease stability.

#### 2.2.2 Targeted AMP Design

**Figure 3:**
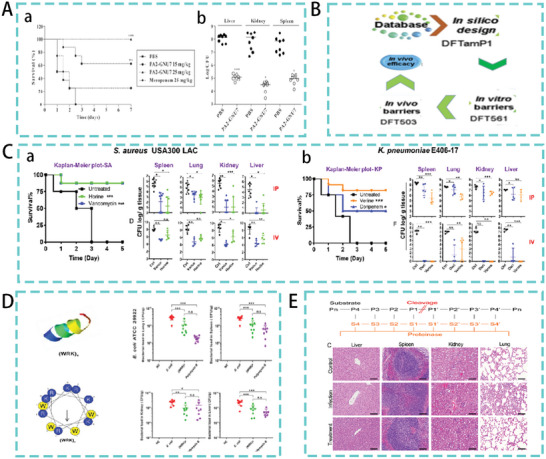
A) Therapeutic efficacy of PA2‐GNU7 in a model of peritonitis. (a) The survival rate of mice. (b) Organ bacterial load in mice. Reproduced with permission.^[^ ^24^ ^]^ Copyright 2020, Elsevier. B) Database‐Based Peptide Design. Adapted with permission.^[^ ^28^ ^]^ Copyright 2019, National Academy of Sciences. C) Therapeutic efficacy of the AMPs horine and verine in a mouse model of peritonitis. (a) and (b) Mice survival data and organ bacterial load in mice. Reproduced with permission.^[^ ^29^ ^]^ Copyright 2020, National Academy of Sciences. D) AMP (WRX)_4_ Helix Wheel and Mimic Diagram. Bacterial load of mouse organs. Reproduced with permission.^[^ ^33^ ^]^ Copyright 2021, American Chemical Society. E) Schematic representation of the protease cleavage site. Organ tissue sections in the peritonitis model. Reproduced with permission.^[^ ^2^ ^]^ Copyright 2020, American Chemical Society.

Introducing targeted peptides into broad‐spectrum AMPs helps convert them into “smart” compounds capable of selectively killing bacteria. Such AMPs maximizes the role of targeted identification of infection sites and targeted killing of pathogenic bacteria while maintaining the homeostasis of the microflora and the health of tissue cells.^[^ ^21^ ^]^ Targeted AMPs can be constructed in various ways and can be used to target lipopolysaccharides (LPS) to kill specific gram‐negative bacteria. For example, Muhle et al. constructed a cysteine *β*‐fold framework to mimic the binding site of LPS and designed the resulting AMP sequence and its derivatives to have a significant bactericidal effect on gram‐negative bacteria antimicrobial activity was 200‐fold higher than that of gram‐positive bacteria.^[^ ^22^ ^]^ The pheromone secreted by bacteria is also an ideal targeting domain, and its fusion with broad‐spectrum AMPs can achieve the purpose of targeting different bacteria. Mao et al. used the *S. aureus* pheromone AgrD1 as the targeting region to fuse with the broad‐spectrum AMP plectasin to construct a targeted AMP against MRSA.^[^ ^23^ ^]^ Targeted AMPs not only have efficient bactericidal effects in vitro, but also show great potential in the treatment of intra‐abdominal infections in vivo. For example, a phage‐displayed peptide library was screened to identify a targeting peptide (PA2) that specifically binds to the OprF porin in *P. aeruginosa*, and a hybrid peptide was constructed by adding the targeting peptide to the potent AMP GNU7. The resulting hybrid peptide PA2‐GNU7 exhibited potent antibacterial activity against *P. aeruginosa*. PA2‐GNU7 was significantly more effective than meropenem in a mouse model of peritonitis induced by drug‐resistant *P. aeruginosa*. A 100% survival rate was observed in the mice treated with PA2‐GNU7 (25 mg kg^−1^). In addition, PA2‐GNU7 treatment significantly reduced the number of bacterial (CFU) present in the liver, kidney, and spleen (**Figure** **3**A).^[^ ^24^ ^]^ Targeted AMP strategies achieve targeted bactericidal effects, and they also shown desirable results in treatment models of bacterial infections. However, the screening and construction of such targeted domains are difficult to design compared to other peptides, and the production costs are high.

#### 2.2.3 Peptide Design Based on Natural Peptide Database

AMPs are rapidly developing in response to the demand for novel antimicrobial agents. To facilitate research in this area, the original database (APD, http://aps.unmc.edu/AP/) was launched by Wang et al. in 2003. APD2 (version 2009) has been updated regularly and extended to APD3. The database currently focuses on AMPs with defined sequences and activities.^[^ ^25^ ^]^ The establishment of the database and the analysis based on the database will help understand the relationship between the characteristics of AMP and the amino acid sequence. Simultaneously, it could enable modern methods to tailor AMP sequences in a targeted manner in response to the emergence of new antibiotic‐resistant bacterial strains and the treatment of bacterial infections.^[^ ^26^ ^]^ For example, studies using “database filtering” and 3D modeling methods to study peptides with 70% hydrophobicity but no hydrophobic patches (>4 hydrophobic amino acids in tandem) and +4 or +5 charges are most likely to be good antituberculosis candidates.^[^ ^27^ ^]^ Similarly, DFTamP1 (Figure 3B), designed based on the antimicrobial peptide database, can effectively kill gram‐positive bacteria. The optimized DFT503, reduces the bacterial load of mouse organs in a mouse model of *S. aureus*‐mediated systemic infection and has the potential to treat bacterial infection in vivo.^[^ ^28^ ^]^ Different to the above‐mentioned methods, Lakshmaiah et al. studied the linear relationship between the average content of arginine (R) and hydrophobic amino acids (Pho) by analyzing more than 3000 AMPs in the antimicrobial peptide database. Based on this R‐Pho relationship, a peptide template was identified and two representative amphiphilic peptides with different structures and activities were designed. Notably, in the peritonitis model (*S. aureus USA300 LAC* and *K*. *pneumonia E406‐17*), the therapeutic effects of the horine and verine peptides were comparable to those of antibiotic treatment. As shown in Figure 3C, all mice in the untreated group died within 3 days. However, peptide‐treated mice achieved a survival rate of over 75% during the 5‐day observation period. In addition, intraperitoneal and intravenous treatment significantly reduced the organ bacterial load in the mice.^[^ ^29^ ^]^ Given the availability of large datasets, researchers can build complex models that tease out patterns and rationales not visible to the human eye fails.^[^ ^26^ ^]^ Effective antibacterial drugs can be developed by fully utilizing database resources. As the optimized sequences may have high sequence homology with host defense peptides, innate immunity may be inadvertently compromised if antimicrobial resistance develops.^[^ ^30^ ^]^

#### 2.2.4 New Sequence Template AMPs Therapy

Research on the stability and structure–function relationship of AMPs has gradually improved over the last 10 years. These studies are widely used to assess the methods for de novo synthetic peptides.^[^ ^31^ ^]^ Combining two critical parameters affecting the activity of AMPs, positive charge and hydrophobicity, AMPs with high activity were designed using through templates. For example, in our previous study, the designed repeat sequence (XXYY)n (where Y is a cationic amino acid, X is a hydrophobic amino acid, and n is the number of repeat units) was successfully used to synthesize short and highly active amphiphilic *α*‐helical peptides.^[^ ^32^ ^]^ Similarly, some studies selected arginine and tryptophan as the positively charged and hydrophobic amino acids of the peptide sequence and designed and synthesized a series of new peptides with repeat sequence (WRX)*_n_* (X represents I, L, F, W, and K; *n* = 2, 3, 4, or 5). Among them, (WRK)_4_ not only has efficient activity in vitro, but also in a mouse peritonitis model, (WRK)_4_ treatment significantly reduced the bacterial load in the lung, spleen, kidney, and liver of infected mouse (Figure 3D).^[^ ^33^ ^]^ A few studies were based on protease‐specific cleavage sites and symmetrical end labeling to develop a highly stable antimicrobial peptide template XX(XCRKPX)*_n_* XX (where *n* = 2, 3, 4, 5; X = I, V). Among them, II‐I_4_‐II (IIICRKPIICRKPIICRKPIICRKPIII‐NH_2_) had an excellent therapeutic effect in *E. coli*‐mediated mouse peritonitis model and significantly reduced inflammatory damage to organs (Figure 3E).^[^ ^2^ ^]^ In combination with the performance of AMPs in experimental studies in vivo, long‐acting peptides are considered as the “golden target” for peptide therapy. Therefore, there is an urgent need to extend the plasma half‐life of peptides to achieve sustained action. The plasma half‐life is mainly caused by rapid and intense renal clearance and proteolytic cleavage during systemic administration. The design of template peptide sequences based on proteolytic cleavage sites has the potential to be more effective in achieving prolonged peptide efficacy in vivo, as well as in the treatment of bacterial infections.

#### 2.2.5 Fatty Acid Modification

**Figure 4:**
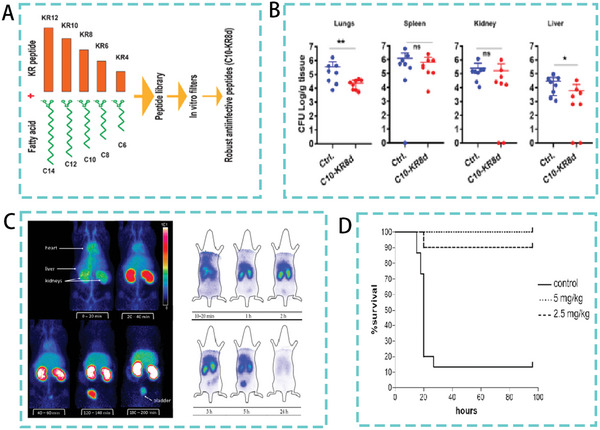
A) Flow chart of the coupling of AMPs with fatty acids of different chain lengths. B) Systemic efficacy of C10‐KR8d in mice infected with *S. aureus USA300 LAC*. Reproduced with permission.^[^ ^35^ ^]^ Copyright 2021, American Chemical Society. C) Biodistribution of d‐Tyr‐danalexin. Reproduced with permission.^[^ ^37^ ^]^ Copyright 2019, CC BY. D) Survival rate of mice treated with SET‐M33D peptide. Reproduced under the terms of the CC BY 4.0 license.^[^ ^39^ ^]^ Copyright 2020, MDPI Publishing.

Owing to high hydrophobicity, fatty acids endow AMPs with additional hydrophobicity, which can significantly improve their membrane‐breaking ability. At the same time, fatty acids can also reduce the degradation of protease, enhance the enzymatic stability of AMPs, and prolong the action time of AMPs in vivo. Studies have shown that the amphiphilic peptide PMAP‐23RI‐Dec can be modified by decanoic acid. It attenuated tissue damage by removing bacteria from the liver and spleen in the *S. aureus*‐induced mouse peritonitis assay. Hematoxylin and eosin (H&E) staining of the liver and spleen sections showed significantly reduced pathological changes in the treatment group.^[^ ^34^ ^]^ A subsequent study combined fatty acids with different chain lengths with AMPs. A lipopeptide library was generated by continuously truncating the smallest AMPs (KR12) of LL‐37 and binding fatty acids with different chain lengths (**Figure** **4**A). C10‐KR8d is an AMP screened in a mouse model of *S. aureus* USA300 LAC‐induced infection. Intraperitoneal injection of C10‐KR8d significantly reduced the bacterial load in the lungs and livers of the mouse (Figure 4B).^[^ ^35^ ^]^

#### 2.2.6 D‐Amino Acid Substitution

Studies have shown that the substitution of D‐amino acids can effectively improve the protease stability and pharmacokinetics of the peptide, and the optimized peptide has the potential to more effectively treat bacterial infections in vivo.^[^ ^36^ ^]^ For example, the AMP D‐Tyr‐danalexin, replaced with D‐type amino acids, not only exhibited higher bacteriostatic activity, but also improved biodistribution in rats, extended retention period, and could remain in the kidneys for longer (Figure 4C).^[^ ^37^ ^]^ Brunetti et al. found that based on the natural peptide Hc‐CATH of sea snake, a derivative of the human HBcARD peptide was successfully designed by D‐amino acid substitution of all arginine residues. This peptide modification strategy significantly increased the half‐life of the DHBcARD peptide an in vitro experimental setting, and there was a significant survival rate between the modified peptide and the original peptide in the *S. aureus*‐mediated mouse peritonitis assay (100% vs 40%).^[^ ^38^ ^]^ Similarly, D‐amino acid modified SET‐M33D exhibited higher activity and stability. In a mouse model of intraperitoneal infection, the survival rate of mice reached 100% after four days of SET‐M33D treatment (Figure 4D).^[^ ^39^ ^]^

### 2.3 Self‐Assembling AMPs Therapy

Self‐assembly refers to the process through which components spontaneously form ordered structures without the intervention of external forces. Owing to their properties, such as size, charge, hydrophobicity, and secondary structure, peptides can self‐assemble into various structures, including nanofibers, nanotubes, nanospheres, and nanogels. Specific biological functions can be directly integrated these such systems through peptide design.^[^ ^40^ ^]^ Self‐assembled peptide‐based strategies have unique properties such as excellent biocompatibility, biodegradability, and flexible responsiveness.^[^ ^41^ ^]^ For example, by appending a pair of glutamic acid and asparagine to the N‐ or C‐terminus of the cleavage peptide, it was found that the N‐terminal modified peptide facilitated the formation of nanofibrils, and the C‐terminal modified peptide formed micelles both nanostructures showed a prolonged action profile and improved serum stability as compared to that in the original peptide.^[^ ^42^ ^]^ Therefore, peptide self‐assembled nanomaterials have great potential for resisting drug‐resistant bacterial infections.^[^ ^43^ ^]^

#### 2.3.1 Lipopeptide Self‐Assembly Therapy

**Figure 5:**
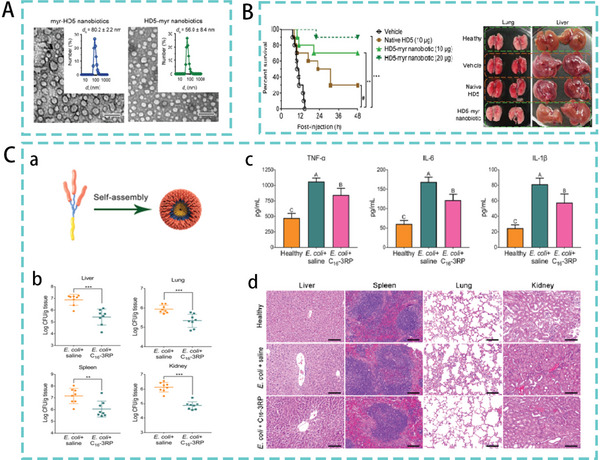
A) TEM images of HD5 and myr‐HD5‐ and HD5‐myr assemblies. B) Therapeutic efficacy of the HD5‐myr nanobiotic in bacterial sepsis. Reproduced with permission.^[^ ^47^ ^]^ Copyright 2018, American Chemical Society. C) (a) Self‐assembled peptide dendritic nanoparticles (SPDN). (b) Bacterial burden in mouse liver, spleen, lung, and kidney in a peritonitis model. (c) Effect of C_16_‐3RP nanoparticles on serum levels of TNF‐*α*, IL‐6, and IL‐1*β* in mice. (d) Histopathological H&E staining of liver, spleen, lung, and kidney tissues. Reproduced under the terms of the Creative Commons CC‐BY license.^[^ ^48^ ^]^ Copyright 2021, The Authors. Published by American Chemical Society.

AMPs can also co‐assemble with fatty acids, and fatty acid‐bound amphiphilic lipopeptides can provide higher amphiphilicity and compatibility, enabling lipopeptides to deliver active substances into cells through endocytosis.^[^ ^44^ ^]^ Therefore, they have an advantage over other amphiphilic peptides. Meanwhile, the self‐assembly of lipopeptides helps present peptide functions at high density on the surface of nanostructures such as fibrils, micelles, and vesicles.^[^ ^45^ ^]^ For example, cholesterol‐modified DP7 readily self‐assembles into stable nanomicelles (DP7‐C) in aqueous solution.^[^ ^46^ ^]^ Human alpha‐defensin 5 (HD5) spontaneously forms nanosphere‐like structures after modification with myristic acid (14‐carbon saturated fatty acid) (**Figure** **5**A). HD5‐myr nanobiotic not only has excellent in vitro bacteriostatic activity, HD5‐myr nanobiotic significantly prolongs the survival of infected mice in a dose‐dependent manner in *E. coli*‐induced mouse models, and the HD5‐treated mice have the same liver and lungs as normal mouse (Figure 5B).^[^ ^47^ ^]^ Our laboratory designed self‐assembled peptide dendrimer nanoparticles using the fatty acid hexadecanoic acid, three arginine‐proline (RP) repeating peptide branches and flexible amino acid linkers (GGG). In a mouse model of *E. coli*‐induced peritonitis, treatment with the nanoparticle C_16_‐3RP, which has excellent antimicrobial capacity and stability, resulted in a significant reduction in bacterial load in liver, spleen, lung, and kidney tissues, as well as a significant reduction in serum pro‐inflammatory cytokine levels (TNF‐*α*, IL‐6, and IL‐1*β*). H&E analyses of mouse liver, spleen, lung, and kidney tissues were also performed as shown in Figure 5C. The C_16_‐3RP nanoparticle treatment largely alleviated or even eliminated these tissue damages compared to the positive control. There was no significant difference in the histological analysis between the two groups.^[^ ^48^ ^]^ In the design of fatty acid‐modified peptide self‐assembly, the increased hydrophobic interactions conferred by fatty acids increase the affinity of the peptide for the surface, thereby increasing its activity.^[^ ^49^ ^]^ It has been proposed that self‐assembled peptides increase the density of amino acid side chains and form a spatial barrier to protect cleavage sites and reduce affinity for proteases. This provides an advantage for the therapeutic efficacy of the peptide in vivo.^[^ ^50^ ^]^ Thus the self‐assembled peptide nanostructure confers high stability and in vivo effectiveness compared to free peptides. Fatty acid‐mediated self‐assembled peptide structures are more effective in treating models of abdominal infection.

#### 2.3.2 AMP Metal Nanoparticle Self‐Assembly Therapy

**Figure 6:**
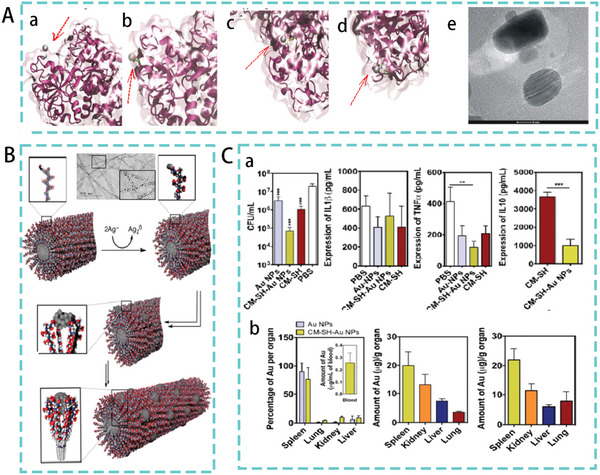
A) Silver cations bind to (a) glutamic acid, (b) aspartic acid, (c) cysteine, and (d) histidine (arrows indicate silver particles). (e) TEM image of the Ag‐LTF nanocomposite. Reproduced with permission.^[^ ^53^ ^]^ Copyright 2016, American Chemical Society. B) Formation of AgNPs on PA nanofibers. Reproduced with permission.^[^ ^54^ ^]^ Copyright 2016, American Chemical Society. C) (a) Antibacterial activity of CM‐SH‐Au NPs in a mouse model of sepsis with pro‐inflammatory (IL‐1b, TNF‐a) and anti‐inflammatory (IL‐10) cytokine levels measured in mouse blood. (b) From left to right, Au levels in organs at 24 h after single and multiple dosing. Reproduced with permission.^[^ ^56^ ^]^ Copyright 2016, Elsevier.

AMPs combined with metal nanoparticles have expanded our understanding of novel bacteriostatic drugs.^[^ ^51^ ^]^ Peptides have a variety of functional groups, including thiol, amino, and carboxyl groups, which chelate precious metals. They all have a strong affinity for Au or Ag atoms, which can bind noble metal atoms more stably, compensating for the shortcomings of metal nanoparticles such as poor stability in aqueous solutions.^[^ ^52^ ^]^ Some studies have combined silver nanoparticles with lactoferrin (LTF). The study found that its binding mode was mainly direct binding to the four amino acids on LTF during adsorption, and the bound nanoparticles showed a characteristic double spherical shape (**Figure** **6**A). Ag‐LTF was confirmed as a fungicide using the disc diffusion method. Its bactericidal activity is much higher than that of LTF.^[^ ^53^ ^]^ Another study reported supramolecular assembly of a novel peptide amphiphile, which peptide amphiphile contains an aldehyde functional group and can nucleate silver metal nanoparticles in water. The system can spontaneously generate monodisperse particles at regular distances along filamentous organic assemblies (Figure 6B). The metal–organic hybrid structure exhibited antibacterial activity with significantly reduced toxicity to eukaryotic cells.^[^ ^54^ ^]^

In addition to AgNPs, AMPs combined with gold nanoparticles (AuNPs) have significant potential for the treatment of bacterial infections.^[^ ^55^ ^]^ AMPs‐conjugated Au NPs also contain high concentrations of AMPs (CM‐SH), controllable size (14 nm), and low polydispersity. In a mouse model of experimental sepsis, CM‐SH‐Au NPs reduced the bacterial concentration in the blood compared with that in animals treated with Au NPs. Furthermore, animals treated with CM‐SH‐Au NPs exhibited lower expression of the anti‐inflammatory molecules IL‐10 and TNF‐*α* than those treated with CM‐SH (Figure 6C). This demonstrating the potential anti‐inflammatory effect of CM‐SH‐Au NPs compared with CM‐SH. In addition, the administration of CM‐SH‐Au NPs did not significantly increase the accumulation of NPs in the organs.^[^ ^56^ ^]^ Previous studies have shown that metal‐based nanoparticles have a nonspecific bacterial mechanism of action (they do not bind to specific receptors in bacterial cells), which not only makes it difficult for bacteria to develop resistance, but also broadens the range of antibacterial activity.^[^ ^57^ ^]^ However, metal nanoparticle‐based AMPs as novel antimicrobial agents for treating bacterial infections, have not shown excellent therapeutic effects in mouse models. Compared with the rest of the previously described self‐assembled peptides, there is still a gap in the reduction of organ bacterial load and inflammatory damage. At the same time the susceptibility of AgNPs to oxidation‐inducing toxicity in mammalian cells and the drawbacks of long‐term retention in vivo remain essential considerations in these trials. Further studies are needed to elucidate the accumulation and biodegradability of metal nanoparticles in vivo.^[^ ^58^ ^]^

### 2.4 Biomaterial‐Based AMP Delivery Therapy

Nanodrug delivery carriers designed through self‐assembly strategies can promote the penetration of antibiotics or antibiotic substitutes, improve drug efficacy, and reduce the risk of drug resistance.^[^ ^50^, ^65^ ^]^ Advances in biomaterials engineering have facilitated the use of AMP to treat of systemic and local infections by reducing the side effects associated with antimicrobial therapy. Mature drug delivery systems, such as surfactants, lipids, and polymer systems,^[^ ^66^ ^]^ inorganic nanomaterials (e.g., metal and metal oxide nanoparticles, (mesoporous) silica, silicon dioxide),^[^ ^60b^ ^]^ and biomolecules, such as chitosan, are used in a wide range of medical applications (**Table** **2**).^[^ ^67^ ^]^ In the study of AMPs, the coupling of AMPs to drug delivery systems increased the duration of action of the peptide and thus its therapeutic effect.

**Table 2:** Key considerations in the construction of delivery systems for AMPs and biomaterial

	Biomaterial	Considerations	Comments	Refs.
AMPs delivery				
Metal nanoparticles	Gold, Silver	Shape, size, spatial arrangement	Metal nanoparticles are durable materials that can accumulate in tissues and should be used with consideration for long‐term toxicity and safety.	[59]
Porous materials	Mesoporous silica, Titanium dioxide	Pore size, surface area, pore structure, charge	Due to well‐defined pores in the nanometer range, drug loading, and release kinetics are broadly controllable; avoiding hydrolysis of antimicrobial peptides by proteases, peptide sealing as well as binding are closely related to void size.	[60]
Polymeric materials	Poly (lactic acid‐glycolic acid copolymer) (PLGA), poly (lactic acid) (PLA)	pH, Pore size	Biodegradable polymer that releases lactic acid to promote angiogenesis and wound healing; configured with AMPs to form a hydrogel.	[61]
	Polyethylene glycol (PEG)	Length, conformation, and linkage type of PEG molecules	PEG‐modified peptides can improve the stability of protease and prolong the action time, but some studies have shown that PEG modification will reduce the activity of peptides; nondegradability.	[62]
	Chitosan	Solubility, uncontrolled pore size	CS is biodegradable, biocompatible, and has low toxicity. CS offers a wide range of applications in tissue engineering, wound healing, and as a drug delivery additive.	[63]
	Polyelectrolytes (poly (ethyleneimine; poly (styrene sulfonate) poly (acrylic acid))	Charge, polyelectrolyte concentration, ionic strength, and pH	Polyelectrolyte complexation provides a versatile route for the design of drug delivery systems for AMP. It reduces peptide toxicity and increases the stability of peptide‐related functional advantages against infection‐related protease degradation.	[64]

#### 2.4.1 Porous Materials and AMP‐Based Conjugation Therapy

**Figure 7:**
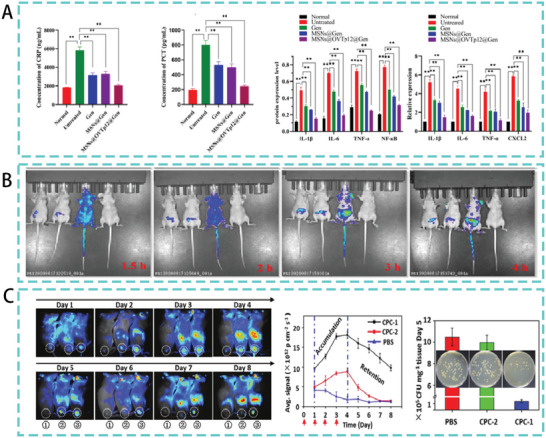
A) In a mouse model of peritonitis, the levels of C‐reactive protein, procalcitonin, and inflammatory factors in mouse serum. Reproduced with permission.^[^ ^73^ ^]^ Copyright 2022, American Chemical Society. B) Biodistribution of AMPs in mice. The mice from left to right were free fluorescein isothiocyanate (FITC), FITC‐labeled N6, FITC‐labeled N6‐COOH‐miniPEG, and the blank control, respectively. Reproduced under the terms of the CC BY 4.0 license.^[^ ^81^ ^]^ Copyright 2022, BioMed Central. C) Accumulation and retention of CPCs and their antibacterial property in vivo. Reproduced with permission.^[^ ^87^ ^]^ Copyright 2017, John Wiley & Sons.

Porous materials are widely controllable in drug loading and release kinetics owing to their well‐defined pores in the nanometer range. For example, the surface area, pore size, form, and surface chemistry.^[^ ^68^ ^]^ Through this, functional advantages can be gained, with increased drug loading, sustained drug release, etc.^[^ ^69^ ^]^ The most widely studied porous materials are mesoporous silica, titanium dioxide, and carbon‐based nanomaterials.^[^ ^70^ ^]^ For example, the delivery of mesoporous silica nanoparticles (MSN) significantly improves the stability of BMP‐2 and reduces its loss during in vivo transport.^[^ ^71^ ^]^ Similarly, Atefyekta et al. synthesized mesoporous titanium dioxide films with different pore sizes (4, 6, and 7 nm) and loaded them with three different antibiotics (vancomycin, gentamicin, and daptomycin), which could be successfully loaded and released from the surface. The results of counting bacterial colony‐forming units showed reduced in bacterial adhesion to the loaded films.^[^ ^72^ ^]^ Ma et al., constructed an AMP delivery system by coupling an ovotransferrin‐derived AMP (OVTp12) with mesoporous silica nanoparticles. In an *E. coli*‐mediated mouse infection model, mesoporous silica‐modified AMPs effectively inhibited the growth of *E. coli* in vivo and significantly reduced C‐reactive protein, procalcitonin, and inflammatory factor levels in mouse serum (**Figure** **7**A). At the same time, the survival rate of treated mice was higher than that of the other peptide‐treated groups. These results show that mesoporous silica‐delivered AMPs have advantages for the treatment of bacterial infections.^[^ ^73^ ^]^ However, the coupling of peptides to their porous materials requires the consideration of various factors. For example, the study by Atefyekta et al. also found that the matching between pore size and peptides is crucial.^[^ ^72^ ^]^ Katharina et al. found in their study that a significant amount of positively charged net peptides could be incorporated into negatively charged mesoporous silica particles. Hence, membrane interactions and the selectivity of peptides and peptide‐carrying nanoparticles are also factors to be considered for the delivery systems of AMPs.^[^ ^74^ ^]^

#### 2.4.2 AMPs Coupled with Polymer Therapy

Peptide‐polymer couples are a class of soft materials consisting of covalently linked proteins/peptides and synthetics/natural polymers. These materials can be used for biological applications such as drug delivery, DNA/gene delivery, and antimicrobial coatings.^[^ ^75^ ^]^ The binding of AMPs to polymers increases their solubility, protects the peptide components from protease degradation and produces more significant binding to avoid rapid renal filtration to prolong circulation in the blood.^[^ ^76^ ^]^ For example, Krista et al. reported that poly (alkyl acrylate) polymers linked with amphiphilic polyetheramines could form stable protective polyelectrolyte nanocomplexes with cationic antimicrobial peptides, such as KSL‐W, providing substantial protection to the antimicrobial peptides and protecting the incorporated peptides from degradation in human plasma.^[^ ^77^ ^]^ In addition, Kizilbey et al. demonstrated that the biological benefits of peptide‐polyelectrolyte complex nanoparticles are broader than those of direct antimicrobial action. These study suggested that peptide‐polymer complexes can effectively activate the immune system without any further adjuvant.^[^ ^78^ ^]^

Polyethylene glycol (PEG) was one of the first, and is still one of the most common polymers bound to AMPs.^[^ ^76^, ^79^ ^]^ In the last few years, polyethylene glycolization strategies have been widely used to increase the lifespan of proteins in blood and to stabilize protein structures.^[^ ^80^ ^]^ Li et al. modified the antimicrobial peptide N6 with linear PEGn of different lengths (*n* = 2, 6, 12, and 24) and found that C‐terminal polyethylene glycolized N6 (*n* = 2, N6‐COOH‐miniPEG) was highly active against gram‐negative bacteria. N6‐COOH‐miniPEG also showed wider biodistribution and prolonged in vivo half‐life in mice (Figure 7B). In addition, N6‐COOH‐miniPEG showed potent bactericidal and anti‐inflammatory effects in a mouse peritonitis model.^[^ ^81^ ^]^ Previous studies in our laboratory have also shown that self‐assembly of PEG‐modified peptides significantly improves pharmacological properties protease stability, and therapeutic index.^[^ ^82^ ^]^ However, it has been suggested that improving the properties of AMP through PEGylation is often at the expense of antimicrobial efficacy and that immunogenicity caused by the accumulation of nonbiodegradable PEG in human organs and tissues is one of the factors limiting its use.^[^ ^83^ ^]^ There is still a lack of research on PEGylated AMPs used in the treatment of bacterial infections in trials, indicating that improving AMPs by PEGylation to treat bacterial infections in vivo remains a challenge.

Chitosan is a naturally occurring polysaccharide that has been extensively investigated for biomedical applications owing to its biocompatibility, biodegradability, mucosal adhesion and anti‐infective activity.^[^ ^84^ ^]^ In addition, this nanocarrier protects the drug from degradation during administration and maintains drug release, thereby increasing half‐life and bioavailability.^[^ ^63b^ ^]^ For example, in a delivery system consisting of chitosan and the natural AMP LL37 (CS/LL37‐NPs), the release of LL37 from CS/LL37‐NPs was almost complete after 5 days according to the in vitro release profile. Furthermore, this delivery system has a prolonged half‐life.^[^ ^85^ ^]^ Similarly, one study coupled chitosan nanoparticles (CNs) to the AMP microprotein J25 (MccJ25). It was demonstrated that the modified coupling compounds were highly active against gram‐negative and gram‐positive bacteria and had stable activity in various thermal and pH conditions.^[^ ^86^ ^]^ CS coupled and encapsulated AMPs can therefore be used as a new weapon against multidrug resistant infections and to increase antimicrobial activity and bioavailability. Qi et al. designed a chitosan‐peptide coupling (CPC) with morphological change in the presence of gelatinase (an enzyme secreted by bacteria). This transformable glycan‐peptide coupling can accumulate and be retained at the site of bacterial infection for a long time. In an in vivo test in mice infected with *S. aureus*, chitosan modification significantly enhanced peptide accumulation and retention at the infection site. It exhibited highly effective antibacterial activity, with and a 10‐fold reduction in bacterial colonies at the infection site (Figure 7C).^[^ ^87^ ^]^ In summary, polymer‐led AMP delivery systems make peptide antimicrobials a great potential for the treatment of bacterial infections. However in vivo evaluation of its effectiveness is still poorly studied and requires further exploration.

## 3 Skin Wound Bacterial Infection

Skin wound healing is a complex and coordinated process consisting of three overlapping phases: inflammatory, proliferative, and maturation of new tissue.^[^ ^88^ ^]^ Disturbances in any of these stages, can result in chronic nonhealing wounds. The main factors contributing to chronic wounds are ischemia and microbial colonization, and wound infection can serve as a bacterial host, leading to high morbidity.^[^ ^89^ ^]^ In response to this problem, AMPs are also an essential tool for treating bacterial infections in skin wounds. Over the past few years, substantial progress has been made in the development and evaluation of AMPs for the treatment of skin infections and for acute and chronic wound healing.

Mouse skin scratch models mediated by *S. aureus* and *A. baumannii* are commonly used to evaluate the efficacy of AMPs in the treatment of skin infections. The mouse skin infection model can be divided into four groups based on the depth of infection as follows: i) subcutaneous infection, ii) intradermal infections, iii) full‐thickness incision in which bacteria are inoculated or wound infections occur in excision wounds, and iv) epidermal infections, in which the skin surface is exposed to bacterial inoculum. A fourth model is often used in studies evaluating the effects of AMPs.^[^ ^90^ ^]^

### 3.1 Optimized Design Based on Natural AMP Sequences

**Figure 8:**
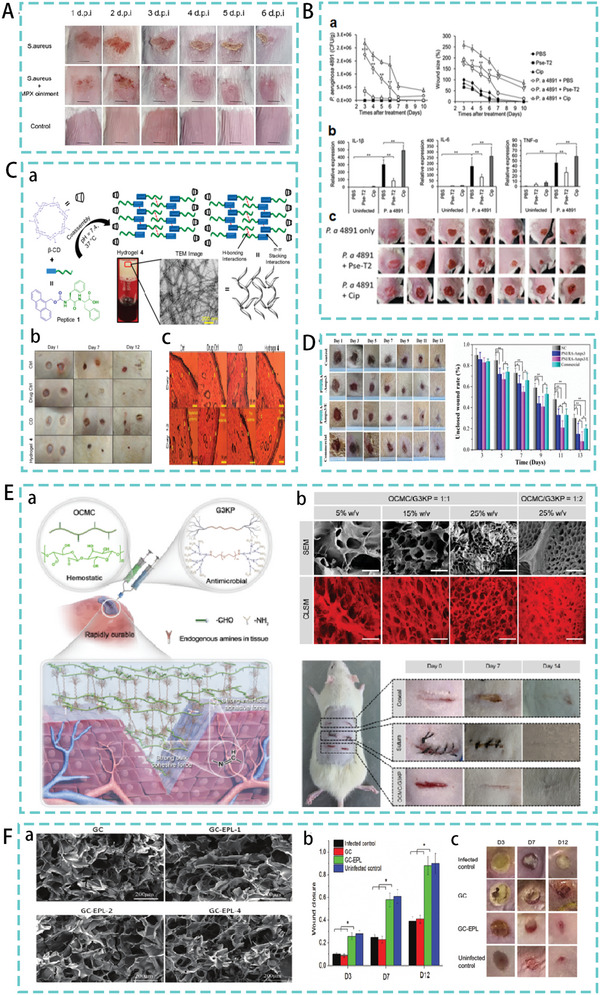
A) Therapeutic effect of MPX in skin infection model. Reproduced under the terms of the CC BY 4.0 license.^[^ ^93^ ^]^ Copyright 2022, Frontiers Media S.A. B) Therapeutic effect of Pse‐T2 in a model of Pseudomonas aeruginosa‐mediated wound infection. Reproduced with permission.^[^ ^96^ ^]^ Copyright 2018, American Society for Microbiology. C) (a) The design process of hydrogel 4 (b) The therapeutic effect of hydrogel 4 in a skin injury model. (c) Histological images of different groups of epithelial tissues. Reproduced with permission.^[^ ^100^ ^]^ Copyright 2020, American Chemical Society. D) Treatment of PNI/RA‐Amps composite hydrogels in a skin infection model. Reproduced with permission.^[^ ^103^ ^]^ Copyright 2022, Royal Society of Chemistry. E) (a) Schematic Illustration of OCMC/G3KP hydrogel bioadhesives. (b) SEM and CLSM images of OCMC/G3KP and promoting wound healing effect of 25% OCMC/G3KP hydrogel. Reproduced with permission.^[^ ^105^ ^]^ Copyright 2020, American Chemical Society. F) (a) Scanning electron microscopy of cryogels. (b),(c) Infection wound healing properties of cryogels. Reproduced with permission.^[^ ^106^ ^]^ Copyright 2020, John Wiley & Sons.

Bacterial wound infections are considered the most severe complications of the wound healing process.^[^ ^91^ ^]^ In addition to intravenous and oral antibiotics, topical antimicrobials are often used to treat infected skin wounds. AMPs have great potential for the treatment of bacterial skin infections.^[^ ^92^ ^]^ For example, MPX, a natural AMP extracted from wasp venom, proved in early trials that MPX could effectively protect mouse from lung damage by *A. pneumonia*. Furthermore, in an *S. aureus*‐mediated mouse scratch infection model, MPX inhibited *S. aureus* colonization, thereby reducing wound size and inflammation, and promoting wound healing (**Figure** **8**A).^[^ ^93^ ^]^ Similarly, Pardaxin, a marine AMP, reduced the number of MRSA bacteria in the injured area and enhanced wound closure in a mouse model of MRSA‐induced skin infections.^[^ ^94^ ^]^

Optimization based on AMPs has also shown potential to treat bacterial infections of the skin. For example, based on the natural AMP (IsCT1‐NH_2_) of scorpion venom, an AMP with a positive charge of 4 was designed and synthesized with multiple amino acid substitutions_._ In a mouse model of skin abrasion induced by *A. baumannii*, the peptide significantly reduced the bacterial load at infected tissue sites.^[^ ^95^ ^]^ Similarly, Pse‐T2 was synthesized using a Lys substitution and truncation design based on the frog‐derived AMP Pseudin‐2. Wounds infected with MDR *P. aeruginosa* treated with Pse‐T2 healed significantly faster than untreated wounds or wounds treated with ciprofloxacin (Figure 8B). In addition, Pse‐T2 can reduce inflammation by inhibiting interleukin‐1, promoting the healing of infected wounds.^[^ ^96^ ^]^ However, the tissue site of infection is usually characterized by high proteolytic activity mediated by bacterial proteases and proteases from human defense cells.^[^ ^97^ ^]^ Therefore, the primary consideration in treating skin infections is the design of AMPs with proteolytic stability. The use of AMPs for chronic wound infections may result in the rapid degradation of the peptide and a corresponding loss of activity. Strategies to improve protease stability of AMPs have been described in detail previously.^[^ ^17a^ ^]^ Second, innovative approaches are needed to treat damaged and difficult‐to‐heal wounds. New strategies are needed to accelerate healing by reducing infections, moisturizing wounds, stimulating healing mechanisms, accelerating wound closure, and reducing scar formation.

### 3.2 AMPs‐Hydrogel Therapy

Hydrogels are the most commonly used drug carriers for open wounds and the combination of hydrogels and AMPs has become an essential means of treating bacterial infections.^[^ ^98^ ^]^ Hydrogels are ideal wound dressings that are widely used to promote wound healing.^[^ ^99^ ^]^ Owing to its porous network structure, the hydrogels can also be used as a drug delivery medium to deliver different drugs to designated sites for slow‐release purposes.^[^ ^100^ ^]^ Peptide‐based self‐assembled hydrogels as well as carrier hydrogels are currently the most widely studied strategies for the treatment of bacterial skin infections.^[^ ^101^ ^]^

#### 3.2.1 Self‐Assembled Peptide‐Based Hydrogels

Peptides can self‐assemble into hydrogels through covalent interactions. In one study, an Amoc (9‐anthracenylmethoxycarbonyl)‐capped dipeptide was designed and synthesized, which self‐assembled into a tough and robust hydrogel owing to the involvement of various noncovalent interactions. The mechanical strength of the self‐assembled peptide‐based hydrogel was adjusted by incorporating equimolar amounts of *β*‐cyclodextrin (CD), resulting in a stable coassembled hydrogel suitable for wound‐healing applications (Hydrogel 4). The nanostructured morphology of the coassembled hydrogels showed highly cross‐linked and entangled nanofibrous networks. In the infection model, the degree of healing after Hydrogel 4 treatment was comparable to that after drug treatment. Histological images of the hydrogel 4 treated group showed significant wound‐healing activity (Figure 8C).^[^ ^100^ ^]^

Self‐assembled AMPs can be combined with other macromolecules, such as polymers and chitosan. This improves the stability and permeability of AMPs and their short half‐lives.^[^ ^102^ ^]^ For example, one study coupled the self‐assembled peptide RADA16 with AMPs and then blended them with the traditional thermosensitive polymer material (PNIPAM) to produce the final hydrogel (PNI/RA‐Amps). In addition, to promote wound healing, peptides with cell proliferative properties were loaded into the hydrogels. The hydrogel was also triggered by body temperature to gelate in situ, resulting in bacterial inhibition and hemostasis during wound healing. Excellent skin healing promoting effect was observed in a mouse skin infection model (Figure 8D).^[^ ^103^ ^]^ Wang et al. prepared injectable hydrogels consisting of polylysine (*ε*‐PL) and carboxymethyl chitosan (CMCS) for medical applications.^[^ ^104^ ^]^ Similarly, Zhu et al. designed a hydrogel consisting of carboxymethyl chitosan (CMC) and a peptide dendrimer AMP (G3KP). The OCMC/G3KP hydrogel had a significant advantage over the other groups in the assessment of wound closure. They have strong tissue integration with the damaged tissue surface, provide rapid hemostasis and exhibit high antimicrobial activity (Figure 8E).^[^ ^105^ ^]^ Hou et al. developed a series of polysaccharide peptide cryogels with excellent antibacterial and hemostatic properties using chitosan (GC) and *ε*‐polylysine (EPL) as the main ingredients. In a mouse skin wound infection model, the GC‐EPL cryogel treatment group had a wound closure rate of over 85% on day 12. The GC‐EPL design was evaluated for its efficacy in promoting skin healing (Figure 8F).^[^ ^106^ ^]^ The new composite hydrogel is composed of biomaterials with different functions and AMPs. Although they have a basic antibacterial effect, they also have the added benefit of stopping bleeding and promoting wound healing, offering a new way of thinking about the treatment of bacterial skin infections.

#### 3.2.2 AMPs‐Carrier Hydrogels

**Figure 9:**
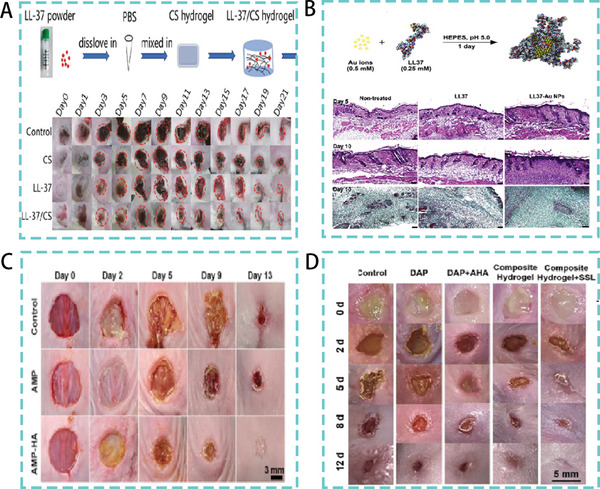
A) LL‐37/CS hydrogel synthesis process. Therapeutic effect in a mouse model of wound infections. Reproduced under the terms of the CC BY 4.0 license.^[^ ^110^ ^]^ Copyright 2020, BioMed Central. B) Schematic diagram of LL‐37 and gold nanoparticle embellishment. Tissue sections in a mice model of skin infection. Reproduced with permission.^[^ ^111^ ^]^ Copyright 2017, Elsevier. C) Therapeutic effect of AMPs in a mice model of *S. aureus* infection. Reproduced with permission.^[^ ^117^ ^]^ Copyright 2021, American Chemical Society. D) Therapeutic efficacy of a HA‐based composite hydrogel composed of AMPs and iron ions in a mice mode. Reproduced with permission.^[^ ^118^ ^]^ Copyright 2022, John Wiley & Sons.

Some AMPs cannot self‐assemble into gels independently but can achieve bactericidal efficacy by deploying into gels to form combined hydrogels.^[^ ^107^ ^]^ The AMP PXL150 was formulated in a hydroxypropyl cellulose (HPC) gel. In a mouse model of infected burn wounds compared to untreated wounds, treatment with 10 mg g^−1^ PXL150 in 1.5% HPC suppressed infection on day 1 and persisted throughout the study. Surviving bacteria in the wounds were reduced by 98% after 4 days of treatment.^[^ ^108^ ^]^ The natural AMP LL‐37 has been found to have a significant effect in promoting trauma angiogenesis and skin regeneration.^[^ ^109^ ^]^ Based on the function of LL‐37, studies have been conducted to encapsulate LL‐37 in chitosan hydrogels. The LL‐37/chitosan hydrogels can effectively deliver LL‐37 peptides to the wound site. with efficient antibacterial and pro‐healing activities (**Figure** **9**A).^[^ ^110^ ^]^ In addition to chitosan, AMPs can also be conjugated to metal nanoparticles for wound delivery. In a large in vitro mouse wound model, LL37‐conjugated gold nanoparticles showed higher pro‐migratory properties and in vivo skin wound healing activity in keratin‐forming cells than soluble LL37 (Figure 9B).^[^ ^111^ ^]^

### 3.3 Combination of AMPs with other Dressings

It has also been suggested that hydrogels are less suitable for exuding wounds and that some coated dressings are suitable for extended drug release and are more clinically appropriate where frequent dressing changes are not required.^[^ ^112^ ^]^ Therefore, in the case of profoundly exuding wounds, combining AMPs with these dressings can achieve better results in treating bacterial infections and promoting healing.^[^ ^113^ ^]^ For example hyaluronic acid (HA), alginate (ALG), poly (lactic acid) (PLA), poly (lactic‐*co*‐glycolic acid) (PLGA), and polyurethane (PU) are among the most widely used polymeric materials for the management and treatment of wounds.^[^ ^114^ ^]^ PU dressings are absorbent dressings that can be clinically used for acute or chronic exuding wounds. The dual‐action wound‐dressing concept was developed by Lin et al. The host defense peptide TCP‐25 was bound to PU. The bound hydrogel targets bacterial infections and the accompanying inflammatory response. In a mouse wound model induced by *S. aureus* TCP‐25 PU showed excellent therapeutic efficacy.^[^ ^115^ ^]^ Similarly, ALG dressings contain a highly absorbent fibrous fleece that limits wound secretion and minimizes bacterial contamination. They are recommended for highly exuding wounds as they can absorb 15–20 times their weight in fluid. For instance, Lin et al. found that AMP (Tet213) was immobilized on ALG, HA, and collagen (COL). The resulting wound dressings exhibited a high degree of swelling, appropriate porosity, mechanical properties. and biodegradability. It has an efficient bactericidal effect on pathogenic bacterial strains while promoting wound healing and epithelial reformation.^[^ ^116^ ^]^ Similarly, Suo et al. designed AMP‐HA composite hydrogels with injectability, high biostability, and enhanced mechanical strength. The AMP‐HA composites showed excellent broad‐spectrum antibacterial activity both in vitro and in vivo. It also promoted wound healing in a mouse model of *S. aureus* (Figure 9C). This provides an effective strategy for treating chronic bacteria infected wounds with antibiotic‐free hydrogel biomaterials.^[^ ^117^ ^]^ Xiong et al. also developed an HA‐based composite hydrogel consisting of AMPs and iron ions, which can photothermally assist and accelerate the healing process of bacterially infected wounds (Figure 9D).^[^ ^118^ ^]^ These studies show that in addition to the inherent antibacterial activity, the multifunctional composite hydrogel also has antioxidant capacity, photothermal effects, and a highly effective healing‐promoting effect. It has significant wound healing potential for bacterial infections.

## 4 Eye Infection

Microbial keratitis is a common cause of ocular pain and visual impairment worldwide.^[^ ^119^ ^]^ The cornea can be infected with a variety of pathogens, including *S. aureus*, *P. aeruginosa*, and *Fusarium*.^[^ ^120^ ^]^ Topical antimicrobial therapy is the standard of care for ocular pathogenic infections compared to intra‐abdominal infections, which are complex, and skin infections, which have high proteolytic activity.^[^ ^121^ ^]^ Several AMPs have shown promise in animal models of keratitis, particularly for the treatment of keratitis infections mediated by *P. aeruginosa*, *S. aureus*, and *Candida albicans*.^[^ ^122^ ^]^ Model building can be roughly divided into two types. The first was the direct infection method, where, after 1 week of feeding, mice were immunosuppressed by daily injection of cyclophosphamide (150 g kg^−1^) for 10 days. Subsequently, the mouse cornea was scratched gently using a needle. Bacterial solution was added to the corneal scratches of each mouse. After inoculation (48 h) of the eyeball, a tough, raised coating was observed on the corneal surface of each mouse, indicating that the model was successfully established. The second was the contact lens‐induced microbial keratitis model in mouse, where contact lenses were inoculated with bacteria to grow a biofilm, and the lenses were placed on a pretreated corneal surface. The contact lenses were removed 12 h after implantation. The effectiveness of peptide treatment was assessed by measuring the bacterial load on the cornea, corneal tissue sections, and clinical scores.

### 4.1 Natural AMPs Therapy

**Figure 10:**
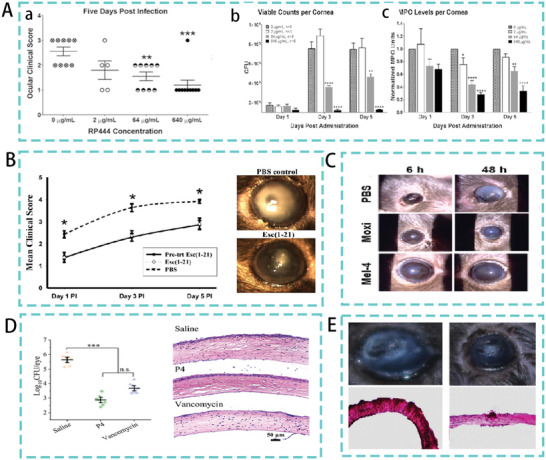
A) (a) Mean clinical scores in RP444‐treated mice keratitis model (b) Bacterial load (c) Level of inflammatory cell infiltration in RP444 topically treated mice keratitis model. Reproduced with permission.^[^ ^123^ ^]^ Copyright 2017, Association for Research in Visual and Ophthalmology. B) Therapeutic effect of Esc (1‐21) in a mice model. Reproduced with permission.^[^ ^124^ ^]^ Copyright 2015, Springer Nature. C) Therapeutic effect of modified bee toxin in a mice model. Reproduced with permission.^[^ ^127^ ^]^ Copyright 2020, American Chemical Society. D) CFU of MRSA after treatment with saline, P4 and vancomycin in a keratitis model. Representative histological analysis of infected mice after antimicrobial treatment corneas (HE staining). Reproduced with permission.^[^ ^122a^ ^]^ Copyright 2022, American Chemical Society. E) From left to right, histology of corneas from 0.7% saline (control), W8 (5, 1 mg mL^−1^) treated mice models, and corneas treated with specific solutions, respectively. Reproduced with permission.^[^ ^128^ ^]^ Copyright 2019, American Chemical Society.

The conventional use of antibiotics for the treatment of infectious keratitis currently faces two major challenges: poor drug penetration and the emergence of antibiotic resistance in microbial strains. AMPs with broad‐spectrum antimicrobial activity and a membrane mechanism of action may address these challenges. A synthetic peptide mimetic of RP444, a human defense peptide, reduced the bacterial load of *P. aeruginosa* infection in a mouse model of keratitis. Furthermore, RP444 treatment significantly reduced clinical scores and a dose‐dependent reduction in inflammatory cell infiltration (**Figure** **10**A).^[^ ^123^ ^]^ Similarly, a study identified the frog skin‐derived AMP esculentin‐1a (1‐21)‐NH_2_ as a candidate for the development of novel topical agents against *P. aeruginosa* keratitis. Esc (1‐21) was administered dropwise at 40 µm to the ocular surface (thrice daily for 5 days postinfection) in a mouse model of *P. aeruginosa* keratitis resulting in a significant reduction in infection (Figure 10B). This study also showed that the designed peptide could disrupt the activity of *P. aeruginosa* biofilms.^[^ ^124^ ^]^ Similarly, the CAP37‐derived peptide 120–146 WH is effective in clearing corneal infections caused by *P. aeruginosa*, in addition to accelerating corneal wound healing.^[^ ^125^ ^]^ However, experimental treatment of bacterial keratitis with AMPs has yielded inconsistent results. The toxic effects of high concentration peptide therapy are also essential factors. For example, the peptide regimen requires multiple doses, with the natural peptide COL‐1 administered every 15 min for the first hour and then every hour for the next 9 h. It is then administered hourly for 10 h on days 2–4.^[^ ^126^ ^]^

### 4.2 Natural AMPs Optimization Therapy

Natural AMPs can be cytotoxic at concentrations required for antimicrobial action and naturally occurring AMPs can be inhibited by high salt concentrations in ocular surface tears. Efforts have been made to overcome these deficiencies by modifying naturally occurring AMPs to provide them with excellent activity and stability. One study systematically replaced the *α*‐lysine residue in bee toxin with an *ε*‐lysine residue, and the modified bee toxin reduced *S. aureus* abundance in a mouse keratitis model (Figure 10C).^[^ ^127^ ^]^ Qian et al. designed and synthesized a series of *β*‐peptide polymers. The best of these polymers showed effective activity against antibiotic‐resistant bacteria, with low acute skin toxicity and low corneal epithelial cell toxicity. A high efficacy was observed in the treatment for MRSA‐induced wound infections and keratitis, which was higher than vancomycin as well (Figure 10D).^[^ ^122a^ ^]^ Similarly, a study was performed on Kunitzin‐RE (an amphibian‐derived bioactivity peptide)‐derived peptide W8, designed by intercepting peptide fragments and single point mutations. W8 showed high cell selectivity and salt tolerance in vitro. In contrast, it showed efficient inhibition of keratitis caused by *C. albicans* infection in mice (Figure 10E).^[^ ^128^ ^]^ The development of AMPs is ongoing and has shown encouraging results in the treatment of eye infections.^[^ ^119^, ^129^ ^]^ Future research should focus on ways to reduce the development of drug resistance while adding a combination of in vitro and in vivo assessments.

## 5 Other Bacterial Infection Types

In addition to the above‐mentioned commonly used animal models of infection used in the study of AMPs, models of osteomyelitis and lung infection also exist. MRSA is a common pathogen in osteomyelitis models. The pathogenic bacterial suspension was injected into the knee joint after drilling the bone ring. Surgical debridement and joint cleaning were performed on day 3 to simulate the surgery. The effect of peptide therapy was evaluated by measuring the bacterial load in the bone marrow and bone. Lung infection models are often induced by *P. aeruginosa* and MRSA. Models can be established in two ways. The first is the inhalation inoculation, in which the strain is placed at the tip of the nostril. Animals were allowed to inhale the inoculum in small droplets and were then returned to their respective cages for recovery and observation. Peptide therapy was administered at 24 and 36 h postinfection. The second was catheter‐mediated lung infection, where two catheters were inserted into the trachea of the mice. Pulmonary infection was induced by intratracheal injection of the therapeutic bacteria. The trial was assessed by lung histological analysis, lung colony counts, and blood inflammatory factor levels to assess the effect of peptide treatment.

### 5.1 AMPs in the Treatment of Bone Infection

**Figure 11:**
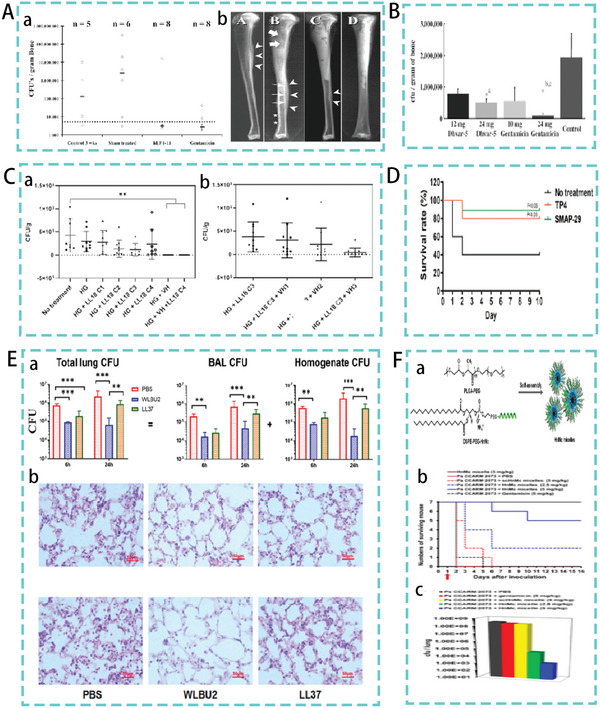
A)(a) Quantitative microbiological analysis of cultured bone homogenates. (b) Representative radiographs of excised tibiae. Reproduced with permission.^[^ ^131^ ^]^ Copyright 2005, American Society for Microbiology. B) Bacterial load in the femur in a model of osteomyelitis. Reproduced with permission.^[^ ^132^ ^]^ Copyright 2004, Oxford University Press. C) Mean bacterial count at the implantation site after 7 days of treatment for MRSA‐induced osteomyelitis. (a) Increasing concentrations of LL18, including empty HG and VH‐loaded preparations (b) Increasing concentrations of VH‐loaded preparations with fixed LL18 concentrations. Reproduced with permission.^[^ ^133^ ^]^ Copyright 2022, John Wiley & Sons. D) Survival rate in mice model. Reproduced under the terms of the CC BY 4.0 license.^[^ ^135^ ^]^ Copyright 2021, Nature Publishing Group. E) (a) Lung bacterial load in a lung infection model. (b) Histological analysis of lung tissue. Reproduced with permission.^[^ ^136^ ^]^ Copyright 2018, Elsevier. F) (a) Chimeric antimicrobial HnMc micelle protocol formulated with DSPE‐PEG‐HnMc and PLGA‐PEG. (b),(c) The mice survival and lung bacterial load after 16 days in a mice model of lung infection. Reproduced with permission.^[^ ^137^ ^]^ Copyright 2020, American Chemical Society.

Osteomyelitis is one of the most common and difficult‐to‐treat infections in orthopedic surgery. Radical surgical debridement with topical antibiotics is the treatment of choice for this condition.^[^ ^130^ ^]^ However, with the development of drug‐resistant bacteria, concerns have arisen regarding this antibiotic treatment. The discovery and advancement of AMPs provided new ideas for the treatment of osteomyelitis. Christopher et al. investigated the therapeutic efficacy of the AMP hLF1‐11 in a model of MRSA‐induced osteomyelitis. hLF1‐11 was incorporated into the Ca‐P bone cement as a viable strategy for treating osteomyelitis. The results of the trial showed a significant reduction in bacterial load in the hLF1‐11 treated group compared to that in the control group. Representative radiographs of the resected tibiae (**Figure** **11**A) showed that animals in the hLF1‐11 treatment group showed no signs of osteomyelitis.^[^ ^131^ ^]^ Similarly, the AMP Dhvar‐5 was incorporated into polymethylmethacrylate (PMMA) beads as a topical drug delivery system. In a rabbit osteomyelitis model, the Dhvar‐5 system significantly reduced the bacterial load in the inoculated femur (Figure 11B).^[^ ^132^ ^]^ However, in these first‐stage treatment studies, AMPs did not kill all the bacteria, which is consistent with the surgical treatment of chronic osteomyelitis. Staging treatment is often required in addition to the first‐stage treatment. The treatment of osteomyelitis infection with a single AMP remains prophylactic, with complete eradication becoming a more important goal. Alexandra et al. developed a topical drug delivery system based on the link between the antimicrobial and regenerative effects in the treatment of osteomyelitis. The system consisted of the AMP LL18 (LLKKK18), vancomycin hydrochloride (VH), and injectable oxydextrin (ODEX)‐based hydrogel. In a clinical MRSA‐induced osteomyelitis model in mice, LL18 exerted an immunomodulatory effect in a dose‐dependent manner as compared to the use of 28 mm VH, and a concentration of 300 µm combined with 483 µm VH eradicated the infection in 70% of individuals and tissue damage (Figure 11C).^[^ ^133^ ^]^ Osteomyelitis treatment has encouraged further research into the ability of AMPs to clear infection while stimulating bone regeneration. Overall, AMPs can be combined with other biocompatible, cost‐effective, and easy‐to‐manufacture biomaterials such as HG as a noninvasive delivery system for osteomyelitis treatment, which is a promising strategy.

### 5.2 AMPs Therapy in Lung Infection

AMPs have great potential for the treatment of lung infections, with a large number of peptides designed to treat lung infections with excellent therapeutic efficacy in mouse models.^[^ ^134^ ^]^ One study screened for the AMP SMAP‐29 and TP4, which were identified as having a preventive effect against pneumonia in mice. TP4 and SMAP‐29 significantly reduced mortality associated with *A. baumannii*‐induced pneumonia by peritoneal or intravenous administration in a mouse model of pneumonia. (Figure 11D).^[^ ^135^ ^]^ Chen et al., designed the AMP WLBU2 consisting of Arg, Val, and Trp only. In the mouse lung infection model assay, WLBU2 significantly (*p* < 0.001) reduced the bacterial load by 10‐ to 100‐fold. In lung tissue sections, the level of inflammatory infiltrate in WLBU2‐treated mice was also significantly reduced compared to that in PBS‐ and LL37‐treated mouse (Figure 11E).^[^ ^136^ ^]^ Notably, WLBU2 was designed based on optimal amphiphilicity using only hydrophobic (Val and Trp) and cationic (Arg) amino acids to minimize amino acid composition diversity. Compared with AMPs, which rely on structural diversity for their action, WLBU2 exhibits superior therapeutic effects in vivo. Park et al. designed a new family of antimicrobial agents, which self‐assembled from a chimeric antimicrobial lipopeptide (DSPE‐HnMc) and amphiphilic biodegradable polymer. This can effectively bind bacterial membranes and kill a broad spectrum of bacteria and biofilms. In the infected lung test, HnMc micelles were administered intravenously 24 h after infection. The results showed that HnMc micelles preferentially accumulated in infected lungs. In contrast, survival data and lung bacterial load at 16 days showed that HnMc micelle‐treated mice had a significantly higher survival rate and a significantly lower lung bacterial load than the other groups. Five of the seven infected mice survived when treated with the 5 mg kg^−1^ dose of HnMc micelles (Figure 11F).^[^ ^137^ ^]^ Encapsulating AMPs in nanostructures can improve AMP stability and activity while reducing systemic toxicity. This combination contains AMPs that are superior to ordinary structures in the treatment of bacterial infections.

## 6 Summary and Outlook

AMP‐based antimicrobials have shown high potential in the treatment of multidrug‐resistant bacterial infections. This article evaluates the therapeutic potential of AMP‐based therapies in animal models of abdominal, skin wound, ocular, and other infections (bone and lung). As the design of biomaterials in combination with AMPs has shown excellent potential for in vivo applications, this review focused on evaluating biomaterial‐based AMP therapies.^[^ ^138^ ^]^

With the development of advanced biomaterials (nanoparticles, chitosan, porous materials, and polymers) coupled with novel AMPs designed with promising bactericidal properties, AMP‐based biomaterial therapies offer significant advantages for the treatment of bacterial infections.^[^ ^139^ ^]^ Compared with single peptides, AMPs that incorporate biomaterials achieve slow release, provide effective local concentrations, reduce off‐target effects and toxicity, improve the stability and activity of the AMPs, and modulate the efficacy of the local microenvironment.^[^ ^140^ ^]^ Additionally, advances in biomaterials engineering will further allow the co‐administration of different classes of antimicrobial agents to maximize therapeutic indices and minimize the development of resistance and other adverse effects.^[^ ^141^ ^]^

Significant and encouraging progress has been made in the research of AMP‐based biomaterial therapeutics. However, the clinical translation of AMP‐based biomaterials has been less successful. Many barriers may hinder the clinical translation of AMP‐based biomaterials, most of which are well known and have been frequently mentioned in recent studies, such as the activity/toxicity paradox and susceptibility to various inhibitory factors (physiological salts, pH, serum, and proteases).^[^ ^142^ ^]^ Future research should address these challenges. First, it is necessary to consider and avoid the binding or hydrolysis effects of these inhibitory factors in new AMP‐based biomaterial design strategies. Various strategies for improving the stability of AMP‐based biomaterials have been discussed in detail in several reviews.^[^ ^17^, ^143^ ^]^ The AMP‐based biomaterials with high stability may be beneficial in the treatment of these biomaterials for various bacterial infections due to their conservative antimicrobial activity.^[^ ^31^, ^144^ ^]^ Second, most AMP‐based biomaterial design or optimization strategies mainly take in vitro antimicrobial activity and toxicity as the primary judgment criteria, while ignoring the current in vivo biocompatibility and therapeutic potential. Thus, although a large database of AMPs has been created and thousands of AMP‐based biomaterials have been modified or newly designed, their clinical translation remains restricted. The correlation between in vitro antimicrobial activity and toxicity and in vivo efficacy and biocompatibility is largely unknown. Biomaterials with high in vitro antimicrobial activity and selectivity are not always efficient in vivo.^[^ ^145^ ^]^ The biomaterials that have the best in vivo therapeutic effect may have already been discarded when the most potent and selective biomaterial was determined by in vitro antimicrobial activity and cytotoxicity for in vivo evaluation in animal models. Therefore, more effective and comprehensive screening methodologies based on in vivo treatment effects and toxicity data should be established in future studies. These screening methodologies are more focused on in vivo toxicity (dose‐effect relationship between AMP‐based biomaterial levels and damage to liver and kidney functions) and in vivo therapeutic effects (survival rate, bacterial load, and histological analysis). Third, more in vivo evaluations and animal models that consider aspects of the clinical environment should be performed, and an AMP‐based biomaterial database that focuses more on in vivo therapeutic effect data should be established. Newly designed or optimized AMP‐based biomaterials based on this database may be more prone to exhibit improved in vivo therapeutic efficacy.^[^ ^85^, ^146^ ^]^

To conclude, the stability and in vivo therapeutic efficacy data of AMP‐based biomaterials should be emphasized in future research. Additionally, while striving to develop novel AMP‐biomaterial‐based bacteriostatic agents, the inherent structures and functions of AMPs and biomaterials should be considered, and parameters need to be tuned to obtain the best performance of the combination. Despite the many barriers that need to be overcome, with the increasing attention and continuous exploration of AMP‐based biomaterials for the treatment of bacterial infections in vivo, the clinical application of AMP‐based biomaterials can expect a bright future.

## Conflict of Interest

The authors declare no conflict of interest.

## Author Contributions

G.L. and Z.L. contributed equally to this work. G.L.: conceptualization, writing‐original draft, writing—review and editing, visualization. Z.L.: conceptualization, writing—review and editing, investigation, visualization. A.S.: writing—review and editing, visualization, supervision, project administration, funding acquisition.
